# Experimental Infection of the *Biomphalaria glabrata* Vector Snail by *Schistosoma mansoni* Parasites Drives Snail Microbiota Dysbiosis

**DOI:** 10.3390/microorganisms9051084

**Published:** 2021-05-18

**Authors:** Anaïs Portet, Eve Toulza, Ana Lokmer, Camille Huot, David Duval, Richard Galinier, Benjamin Gourbal

**Affiliations:** 1 IHPE, University Montpellier, CNRS, Ifremer, University Perpignan Via Domitia, 66860 Perpignan, France; ap2133@cam.ac.uk (A.P.); eve.toulza@univ-perp.fr (E.T.); camillehuot86@gmail.com (C.H.); david.duval@univ-perp.fr (D.D.); richard.galinier@univ-perp.fr (R.G.); 2 Laboratory of Eco-Anthropology UMR 7206 CNRS-MNHN-Paris 7, 75005 Paris, France; ana.lokmer@mnhn.fr

**Keywords:** microbiota, bacteria, *Biomphalaria* snail, *Schistosoma* infection, immune response, dysbiosis

## Abstract

Host-parasite interaction can result in a strong alteration of the host-associated microbiota. This dysbiosis can affect the fitness of the host; can modify pathogen interaction and the outcome of diseases. *Biomphalaria glabrata* is the snail intermediate host of the trematode *Schistosoma mansoni*, the agent of human schistosomiasis, causing hundreds of thousands of deaths every year. Here, we present the first study of the snail bacterial microbiota in response to *Schistosoma* infection. We examined the interplay between *B. glabrata, S. mansoni* and host microbiota. Snails were infected and the microbiota composition was analysed by 16S rDNA amplicon sequencing approach. We demonstrated that the microbial composition of water did not affect the microbiota composition. Then, we characterised the *Biomphalaria* bacterial microbiota at the individual scale in both naive and infected snails. Sympatric and allopatric strains of parasites were used for infections and re-infections to analyse the modification or dysbiosis of snail microbiota in different host-parasite co-evolutionary contexts. Concomitantly, using RNAseq, we investigated the link between bacterial microbiota dysbiosis and snail anti-microbial peptide immune response. This work paves the way for a better understanding of snail/schistosome interaction and should have critical consequences in terms of snail control strategies for fighting schistosomiasis disease in the field.

## 1. Introduction

*Biomphalaria glabrata* is a freshwater snail (Lophotrochozoa, Planorbidae), living in inter-tropical regions of Latin America, in rivers, ponds, waterways and other freshwater environments. *B. glabrata* snails have important medical and epidemiological impacts due to their role as the main intermediate host of *Schistosoma mansoni* (Lophotrochozoa, Platyhelminthes, Trematoda), the agent of intestinal schistosomiasis. Schistosomiasis is the second most widespread human parasitic disease after malaria, affecting over 200 million people worldwide and causing 200,000 deaths annually [1]. The gonochoric *Schistosoma* adult parasites mate in the human host venous system. Female worms produce eggs that cross endothelial mesenteric vessels and intestinal epithelium to reach faeces and finally the aquatic environment. Once in the water, eggs hatch and release miracidia, the free-living snail-infective parasite stage. At the following step of the life cycle, the miracidium needs to infect the freshwater snail *B. glabrata*. Intensive asexual multiplication in the snail tissues leads to the continuous production of hundreds of generations of cercariae, the free-living human-infective parasite stage.

At present, there is no effective vaccine against schistosomiasis and the treatment relies on a single chemotherapeutic treatment, Praziquantel [2], against which resistance has been observed [3]. Molluscicides have been also used to impair *Schistosoma* transmission in the field [4]. However, the dramatic effects of molluscicides on natural environments prompt us to seek new ways to prevent and/or control this disease in the field [5].

In this actual context, it is important to find a new angle to decrease schistosomiasis in the field. Several studies have shown that the microbiota interacts with pathogens and/or host immunity in invertebrates. For example, the midgut microbiota of the mosquito *Aedes* sp. elicits a basal immune activity of the immune system [6] mostly by activating Toll pathways [7]. In *Anopheles gambiae*, some members of its microbiota can limit malaria transmission, by inducing a wide antimicrobial immune response [8]. More precisely, bacteria of the genus *Enterobacter*, found in the mosquito microbiota, were shown to produce reactive oxygen species (ROS) that inhibit *Plasmodium* development [9]. Furthermore, the endosymbiotic bacteria *Wolbachia* induce in *Anopheles stephensi* the expression of immune genes, like TEP1 (Thioester-containing protein 1), LRIM1 (Leucine-Rich Immune Molecule 1) or defensin 1 [10]. In *Drosophila*, the bacterial microbiota is necessary to produce the Pvf2, a PDGF/VEGF-like growth factor that restricts enteric viral infection [11]. The microbiota may also play a role in immune priming. For example, in *Anopheles gambiae*, hemocyte priming during the *Plasmodium* infection is naturally induced by the gut microbiota, whose absence results in more severe infections and re-infections [12]. Similarly, the gut microbiota is necessary for immune priming and efficient response against *Bacillus thuringiensis* in *Tribolium castaneum* [13]. Finally, host immunity can influence the tolerance or control of the microbiota. As an example in the tick *Ixodes scapularis*, the protein PIXR, secreted by the tick’s gut, inhibits bacterial biofilm formation and supports gut eubiosis (healthy gut microbiota), and interestingly its inactivation leads to dysbiosis. This change in gut microbiota facilitates the colonization by *Borrelia burgdorferi*, the Lyme disease agent [14]. In *Drosophila,* the intestinal homeobox gene Caudal regulates the commensal-gut mutualism by repressing nuclear factor kappa B-dependent antimicrobial peptide genes [15]. In Hydra, the innate immune sensors and effectors protect not only against pathogens but also control microbiota homeostasis [16]. Moreover, host-specific antimicrobial peptides [17,18] and a rich repertoire of pattern recognition receptors [19] are involved in maintaining homeostasis between the host and the resident microbiota. Similarly, bactericidal permeability-increasing proteins (BPIs) shape bacterial communities in the light organ of the squid Euprymna scolopes and prevent their spill over to other tissues [20]. All these studies showed strong reciprocal interactions between host immunity, microbiota and potential pathogens and demonstrate that microbiota dysbiosis (i.e., a functional and/or a composition alteration of the host-microbiota resulting in an imbalance of host-microbial communities [21,22]) may change host susceptibility/compatibility towards pathogens. Thus, we propose that a better understanding of interactions between *Schistosoma mansoni, Biomphalaria glabrata* snails and its associated microbiota might represent an interesting approach in searching for new strategies of Schistosomiasis control.

**Figure 1:**
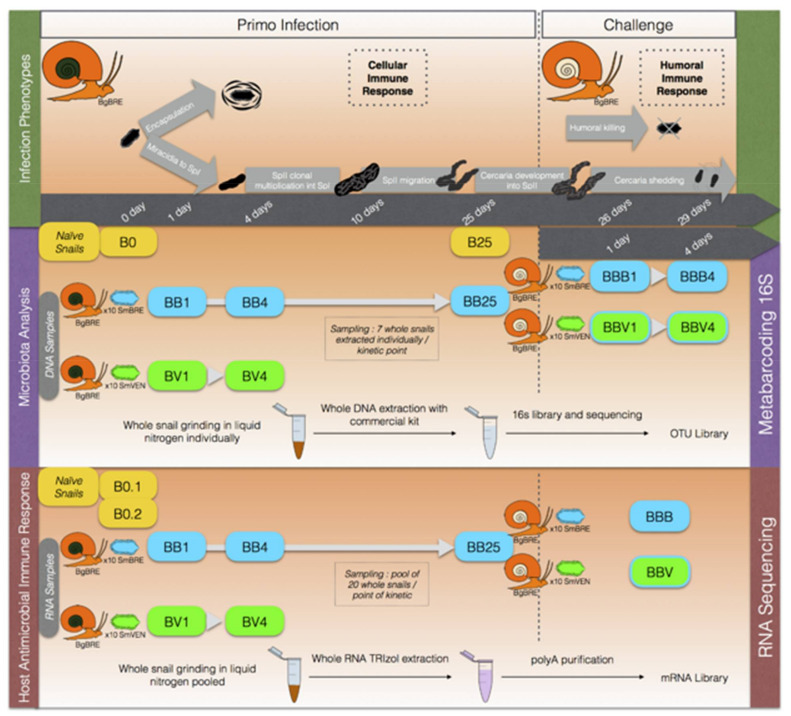
Experimental protocol.

In this context, we have conducted in the last decade numerous studies on immunological interactions between the snail and the parasite [23,24,25,26,27,28]. We have demonstrated that the nature of the snail immune response depends on the strain/species of host or of parasite considered [23,24,25,26,27,28]. Depending on both, the host and the parasite intrinsic capacities, the fate of the interactions can have two possible outcomes: the parasites either (i) develop normally in snail tissues (compatible interaction) or (ii) are encapsulated by haemocytes (the snail immune cells) (incompatible interaction) [24,29,30] (Figure 1). Moreover, totally different immunobiological processes have been described when comparing sympatric versus allopatric interactions, immunosuppression occurs in sympatry while a strong immune response is activated in allopatry [31]. Finally, an "immune shift" consisting of a transition from a cellular immune response toward a humoral immune response has been described when considering the innate immune memory process in *B. glabrata* snails following repeated *S. mansoni* infections [32] (Figure 1). Knowing the close bond between host immunity and microbiota communities, the diverse immunobiological responses described above would be expected to have strong impacts on the dynamics, diversity and composition of the *B. glabrata* associated microbiota during *S. mansoni* infection.

However, in the present host-parasite model, only a few studies examined the bacterial microbiota of *Biomphalaria glabrata* so far [33,34]. The study of aerobic heterotrophic cultivable flora of 200 snails [33] revealed four predominant bacterial genera including *Pseudomonas*, *Acinetobacter, Aeromonas* and *Vibrio*. A few years ago, a study identified six additional bacteria genera: *Citrobacter, Cupriavidus, Rhizobium, Stenotrophomonas, Klebsiella* and *Sphingomonas* in *B. glabrata* cultured bacterial microbiota analysed using 16S rRNA gene sequences [34]. More recently the diversity and abundance of bacterial microbiota within the haemolymph [35] or whole snails [36] of *Biomphalaria* snails originating from different localities or laboratory populations have been also investigated using Illumina MiSeq sequencing of the bacterial 16S rDNA. In addition, it has been demonstrated that the diversity and composition of bacterial microbiota differed between resistant and susceptible *Biomphalaria* to *Schistosoma* infection, with differences in relative abundances of *Gemmatimonas aurantiaca* and *Micavibrio aeruginosavorus*. To our knowledge, there is just one paper that reported the effect of helminth infection (*Angiostrongylus cantonensis*) on the microbiota of the fresh-water (*B. glabrata*) and the terrestrial (*Phyllocaulis soleiformis*) snails. This paper focused exclusively on gut microbiota by analysing faecal DNAs by 16S rDNA sequencing recovered from control and infected snail groups. Interestingly, the microbiota of the two snail species behaves totally differently following *A. cantonensis* infection, with no significant changes observed for *P. soleiformis* but a strong impact on microbiota composition and diversity in *B. glabrata* [37].

These observations support the potential link between snail compatibility, parasite infections and the community composition of snail-associated microbiota [38]. Nonetheless, no studies so far have examined changes in bacterial microbiota of *Biomphalaria* snails following *Schistosoma* infections.

Taking into account all the peculiarities of this system, the present study aimed to investigate the variation of microbiota in snail populations as well as to assess the influence of varying immune response against *Schistosoma* on the host microbiota composition and dynamics. To reach this goal, we first investigated the influence of water microbial communities on snail microbiota, and we characterized the bacterial microbiota of naive *Biomphalaria glabrata* snails using 16S rDNA amplicon sequencing. Then, we analysed changes in microbiota composition, (i) following primary infections with sympatric or allopatric *S. mansoni* parasite isolates displaying the same prevalence and intensity phenotypes but which caused, respectively, immunosuppression or activation of the immune response as previously described by Portet and collaborators [31]; or (ii) following secondary challenges with homologous or heterologous parasite strains. As snail immune response strongly differs between such diverse snail/parasite combinations, we concomitantly analysed, using an RNAseq approach, the snail immune response. Finally, we concluded on the relationship between the contrasted *B. glabrata* immune response profiles and the observed changes in the composition and diversity of microbiota communities.

## 2. Materials and Methods

### 2.1. Ethical Statements

Our laboratory holds permit # A66040 for experiments on animals, which was obtained from the French Ministry of Agriculture and Fisheries and the French Ministry of National Education, Research, and Technology. The housing, breeding and care of the utilized animals followed the ethical requirements of our country. The experimenter possesses an official certificate for animal experimentation from both of the above-listed French ministries (Decree # 87–848, 19 October 1987). The various protocols used in this study have been approved (june 2017) by the French veterinary agency of the DRAAF Languedoc-Roussillon (Direction Régionale de l’Alimentation, de l’Agriculture et de la Forêt), Montpellier, France (authorization # 007083).

### 2.2. Biological Material

#### 2.2.1. Comparison of Snail Microbiota and Water Microbial Composition

To determine the snail’s bacterial microbiota composition and compared it with water microbial composition, we chose 7 different snail strains or species according to their phylogenic distances [36]. Four strains of *Biomphalaria glabrata* were used, one from, Guadeloupe (*B. gla GUA*), and three from Brazil (*B. gla BAR B. gla BRE and B. gla BS90*) originating from Belo Horizonte, Recife and Salvador, respectively. *B. glabrata BS90* is a strain naturally resistant to most of the *S. mansoni* strains from both the new and old world [39]. Finally, we tested also another species of *Biomphalaria*, namely *B. pfeifferi* originating from Oman (*B. pfe OMA*) and two other snail species, one from *Planorbinae* family, *Planorbarius metdjensis* (*P. met*) and a *Planorbidae* non-*Planorbinae* species, *Bulinus truncatus* (*B. tru*) originating from Salamanca and Almeria (Spain) respectively.

All strains were reared in the same conditions; each strain was maintained in separate tanks with pond water (coming from a freshwater borehole), at a constant temperature of 26 °C and fasted 24 h before sampling. Snails were collected and kept individually for sequencing. The tank water was pre-filtered using a 45 μm sieve. Then, pre-filtered water was filtered on three successive membrane filters with porosities of 10, 0.8 and 0.2 μm using a filter unit connected to a vacuum pump. The membranes were then flash-frozen in liquid nitrogen and kept at −80 °C until DNA extraction. The DNA was extracted from individual snails and membranes and 16S rDNA amplicon sequencing was performed to determine the associated bacterial community composition for each snail strain and the corresponding water tanks.

#### 2.2.2. Snail Bacterial Microbiota Study Following *S. mansoni* Infections

A specific snail host is infected by either a sympatric strain or an allopatric strain of parasite. Such sympatric and allopatric interactions were chosen while they resulted in totally opposite immunobiological processes in snails: (i) strong immunosuppression was observed in sympatry and (ii) activation of cellular immune response was observed in allopatry [31]. Therefore, we used the albino Brazilian strain of *Biomphalaria glabrata* (BgBRE) and two strains of the trematode parasite *Schistosoma mansoni*: a Brazilian strain (SmBRE, for sympatric infection) and a Venezuelan strain (SmVEN, for allopatric infection). BgBRE and SmBRE strains originate from the locality of Recife, Brazil; the SmVEN parasite strain was recovered from the locality of Guacara, Venezuela. All host and parasite strains were maintained in the laboratory on their respective sympatric snail hosts (SmBRE on BgBRE and SmVEN on BgVEN). The snails were reared at a constant temperature of 26 °C and fed only with lettuce every 3 days. Before starting the experiment the snails were recovered from several rearing tanks and kept together before infections. After the infections, snails were kept in different tanks (in the same room) with pond water (coming from a freshwater borehole) being mixed between all the experimental tanks and the same procedure was conducted during the time course of the study.

#### 2.2.3. Experimental Infections

To decipher the inter-individual structure of snail microbiota as well as investigate the influence of different snail immune stimulations on its microbiota structure and dynamics, we applied a two-step experimental infection protocol (Figure 1).

Briefly, BgBRE snails were primarily infected with one of the two parasite strains (SmBRE sympatric or SmVEN allopatric). The snails were then sampled 1 and 4 days following primary infection by both parasite strains and 25 days after primary infection for SmBRE infection condition. Then, BgBRE primary infected snails with SmBRE were secondary challenged (25 days after the primary infection) with SmBRE or SmVEN parasite strains to correspond to homologous or heterologous secondary challenges respectively. The secondary challenged snails were then sampled at 1 and 4 days following the secondary challenge (Figure 1). For all experimental infections, the snails were individually exposed for 3 h to 10 miracidia in 5 mL of pond water (coming from the same water tanks used for the rearing), thereafter snails were returned to the water tanks and separated according to the parasite strain and infection conditions. The naive snails are also individually put in water during the same time, to expose naive snails to exactly the same procedures as the infected ones.

#### 2.2.4. Infections and Sampling for Bacterial Microbiota Analysis

We performed a primary infection of 63 BgBRE snails with either SmBRE (sympatric parasite, 49 snails) or SmVEN (allopatric parasite, 14 snails) strains. Then, 25 days after the primary infection, we secondary challenged a subset (28 snails) of SmBRE-primary infected snails with either SmBRE (14 snails) (homologous secondary challenge) or SmVEN (14 snails) (heterologous secondary challenge).

To examine the effect of primary infection on the snail microbiota, we sampled 7 whole snails for each infection combination on day one (named BB1 and BV1; the first letter refers to the *Biomphalaria* strain BgBRE and the second letter refers to the origin of *S. mansoni* strains used for primary infection, SmBRE or SmVEN), on day four (BB4 and BV4), and on day twenty-five (BB25, no SmVEN-infected snails were sampled on day 25) after the primary infection. This BB25 sample is used as a control for microbiota changes observed following secondary challenge infections. Thus, we sampled 7 whole-snails on day one after the secondary challenge (BBB1 and BBV1; the third letter refers to the origin of the *S. mansoni* strain used for the secondary challenge, SmBRE or SmVEN) and on day four after the secondary challenge (BBB4 and BBV4). In addition, we used 6 naive snails collected at the beginning of the experiment (B0) and 6 naive snails collected at the time of the secondary challenge (i.e., day 25, named B25) as controls to assess the variability and stability of the BgBRE bacterial communities in our breeding and rearing laboratory conditions. The naive snails were kept in different tanks but with shared water with all the other infected snails. All snails (naive or infected) were not fed 24 h before the DNA extraction for 16S amplicon sequencing.

#### 2.2.5. Infection and Sampling for Host Antimicrobial Immune Response

We performed a primary infection of 180 BgBRE snails with either SmBRE (sympatric, 140 snails) or SmVEN (allopatric, 40 snails). Then, 25 days after infection, we secondary challenged a subset (80 snails) of SmBRE-infected snails with either SmBRE (40 snails) (homologous secondary challenge) or SmVEN (40 snails) (heterologous secondary challenge) [31].

To study the primary infection transcriptomic immune response of the snail, we took a pool of 20 snails for each infection condition at day 1 (BB1 and BV1), day 4 (BB4 and BV4), and day 25 (BB25) after primary infection. Then after the secondary challenge with SmBRE or SmVEN, we sampled 20 snails in each condition on day 1 and 4. Finally, samples of day 1 and day 4 were mixed together into a single sample referring to BBB or BBV. In addition, we used 2 pools of 30 naive snails (referring to as B0.1 and B0.2) to establish the transcriptomic profile of uninfected control snails [31].

### 2.3. Extraction and Sequencing

#### 2.3.1. DNA Extraction and 16S rDNA Sequencing

Immediately after sampling, snail shells were cleaned with alcohol and removed, whole snails were then frozen in liquid nitrogen and grounded. The total DNA was extracted with DNeasy Blood and Tissue Kit (Qiagen, Courtaboeuf, France) according to the manufacturer’s protocol. The DNA quantification was performed by Qubit 2.0 Fluorometer, using dsDNA Assay kit (ThermoFisher, Waltham, USA). Individual 16S rDNA amplicon libraries were generated using the 341F (CCTACGGGNGGCWGCAG) and 805R (GACTACHVGGGTATCTAATCC) primers targeting the variable V3-V4 loops [40]. Paired-end sequencing with 250 bp read length was performed on the Illumina MiSeq sequencing system (Genome Québec, Montréal, QC, Canada) using the v2 chemistry according to the manufacturer’s protocol.

#### 2.3.2. RNA Extraction and Transcriptomic Sequencing

Immediately after sampling, snail shells were cleaned with alcohol and removed, and then snails were pooled according to infection type. Total RNA was extracted using TRIZOL (Sigma Life Science, Saint Quantin Fallavier, France) according to the manufacturer’s instructions. For BBB and BBV equimolar amounts of RNA extracted from molluscs challenged at both 1 and 4 days were mixed together into a single sample. After library preparation (llumina^®^ Stranded Total RNA Prep), cDNAs were sequenced in paired-end 72-bp read lengths, using the mRNA-Seq kit (QuiaGen) for transcriptome sequencing on Illumina Genome Analyzer II (MGX-Montpellier GenomiX, Montpellier, France).

### 2.4. Microbiota Analysis

#### 2.4.1. Data Analysis of 16S Sequences

For the 16S analysis, we performed an OTUs (Operational Taxonomic Unit) analysis. The FROGS pipeline (Find Rapidly OTU with Galaxy Solution) implemented on a galaxy instance (http://sigenae-workbench.toulouse.inra.fr/galaxy/, accessed on March 2019) was used for data processing [41]. Briefly, paired reads were merged using FLASH [42]. After denoising and primer/adapters removal [43], de novo clustering was done using SWARM, which uses a local clustering threshold, with aggregation distance d = 3 after denoising. Chimeras were removed using VSEARCH [44]. We filtered out the singletons and performed taxonomic assignment using Blast+ against the Silva database (release 128).

All statistical analyses were done using R v3.3.1 (R: a language and environment for statistical computing, 2008; R Development Core Team, R Foundation for Statistical Computing, Vienna, Austria [http://www.R-project.org, accessed on July 2018). We used the phyloseq R package for community composition analysis [45] to infer alpha diversity metrics as well as beta diversity (between-sample distance). Beta diversity was examined by Principal Coordinate Analysis (PCoA) using the Bray–Curtis distance matrices. We performed a Mann–Whitney U test (*p* < 0.05) to compare alpha diversity and one-way PERMANOVA with a Benjamini–Hochberg post-hoc to analyse beta diversity between the experimental groups. To analyse the phylum dynamic during infections we used Mann–Whitney test. For all analyses, the threshold significance level was set at 0.05.

#### 2.4.2. Analysis of Core-Microbiota

We defined the core-microbiota as the set of bacterial families that were present in 100% of the naive individuals excluding unknown or multi-affiliations at higher taxonomic ranks (s S1). We then used the abundances of OTUs belonging to these families to examine the composition of the core microbiota.

To check if the core microbiota was affected by infection, we compared the abundance of core families between infected snails and naive conditions with a one-way PERMANOVA with a Benjamini–Hochberg post-hoc correction. Moreover, a frequency test was performed to determine which specific families were affected during infection. The number of significantly differentially represented families at each sampling day (1, 4 and 25 days after primary infections and 1, 4 days after secondary challenges) was calculated to assess the temporal variability during the course of infection.

### 2.5. Transcriptome Analysis of Antimicrobial Immune Response

#### 2.5.1. Antimicrobial Response

An antimicrobial transcriptome was built from transcripts known to be involved in *Biomphalaria* immune response against bacteria (antimicrobial peptides: biomphamacin or antimicrobial proteins: LBP/BPI and achacin; see Table S2 for details). The full-length sequences of these transcripts were recovered from GenBank and the *Biomphalaria* genome [46] and joined in a subset that represents the antimicrobial transcriptome of *B. glabrata*. This antimicrobial transcriptome was then concatenated with a de-novo assembled transcriptome of *Biomphalaria* available in our laboratory (see [25,32,47] for details) and uploaded on the Galaxy server. Before concatenation, a blastn (70% identity and 90% coverage) was conducted to identify redundant transcripts across the transcriptomes. Redundant transcripts were then discarded using CDhitEst to avoid mapping errors and bias in read counts when using Bowtie2.

#### 2.5.2. Differential Expression Analysis

High-quality reads (Phred score > 29) were aligned to the concatenated transcriptome using Bowtie2 (v.2.0.2), which was run locally on a Galaxy server. The DESeq2 (v2.12) R package was used to identify differential expression levels between uninfected (B0.1 and B0.2) and infected conditions (adjusted *p*-value < 0.05).

## 3. Results

### 3.1. Specificity of Snail Microbiota Compared to Water Microbial Communities

Environmental abiotic conditions can affect microbiota composition. For such aquatic organisms, water is considered one of the potential drivers of individual microbiota composition. Herein we tested whether the same maintenance conditions would lead to a homogeneous composition of the bacterial communities between snail strains. We selected 6 Planorbinae family members: 4 *Biomphalaria glabrata*, 1 *Biomphalaria pfeifferi* and 1 *Planorbarius metdjensis* and 1 non-Planorbinae species, *Bulinus truncatus*. We performed a 16S amplicon sequencing on individual whole snails and membranes used to filter the tank waters for each snail strain.

The hierarchical clustering revealed a strong similarity between the water membrane bacterial community compositions whatever the tank (Figure S1A). The same result was obtained using MDS (Figure S1B). The water membranes of *Planorbarius metdjensis* and *Bulinus truncatus* were relatively separated from the water membranes of *Biomphalaria* snails and closer to their respective associated snail species (Figure S1B). These results revealed that the bacterial communities of the water would tend to become more similar to the microbiota of the molluscs, potentially due to the faeces released by the snails in the water tanks. Thus it seemed that the snails impact the water microbial composition but that the water did not influence the snail microbiota.

Moreover, recently we demonstrated using common garden experiments that the specificity of the mollusc strain/species microbiota is higher than the effect of rearing conditions. When individuals from different strains/species were reared together in the same water tank, every individual of each strain/species maintained a specific bacterial microbiota being moreover indicative of a phylosymbiosis pattern [36].

Based on these observations and results (Figure S1), we can conclude that the bacterial microbiota was highly specific with a limited inter-individual variation for each host strains or species considered, the host genetic/physiology/metabolism/phylogeny highly constraining microbial composition, and thus we concluded that the rearing conditions (i.e., water microbial composition) would not affect bacterial microbiota composition.

### 3.2. Characterization of Healthy B. glabrata Microbiota

To examine the steadiness of naive BgBRE snail microbiota along the time course of the experiment, first, we investigated the microbiota diversity and composition in BgBRE naive snails recovered at day 0 (B0) and 25 (B25) of the experiment. We found no significant differences between the B0 and B25 snails in any of the alpha diversity indices (Tables S3 and S4).

**Figure 2:**
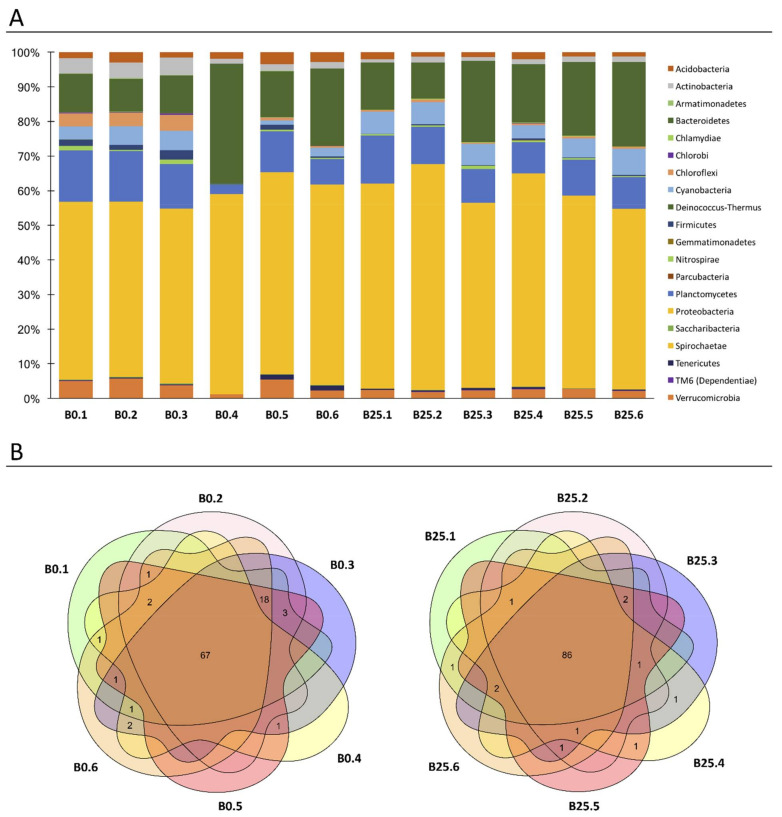
*Biomphalaria glabrata* microbiota characterization: *Biomphalaria* bacterial microbiota of six naive snails recovered at the starting of the experimentation (B0.1; B0.2; B0.3; B0.4; B0.5 and B0.6) and 6 naive snails recovered 25 days after the starting of the experimentation (B25.1; B25.2; B25.3; B25.4; B25.5 and B25.6) were analysed. (**A**). Phylum level composition of the 20 most abundant OTUs phyla among the 12 naive snails. (**B**). The Venn diagram represents the number of the 97 OTUs families, which shared between the 6 naive snails at B0 (left Venn diagram), and between the 6 naive snails at B25 (right Venn diagram).

Naive snails showed little inter-individual variability and a stable composition at the phylum level over time (Figure 2A, Tables S3 and S4), with Proteobacteria, Bacteroidetes, Cyanobacteria and Planctomycetes phyla being the most represented (Figure 2A). Moreover, the bacterial microbiota of naive BgBRE snails displayed considerable temporal and inter-individual stability at the family level (Figure 2B). The fact that naive snail microbiota composition and diversity did not vary over time (Figure 2) could be related to the laboratory rearing conditions and laboratory environmental abiotic factors (water composition, temperature, pH, food) that were tidily controlled.

In terms of composition, we observed that 67 (69%) and 86 (89%) out of 97 identified families were shared by all individuals of B0 or B25 naive snails respectively (Figure 2B). Those results were used to determine the core microbiota. We defined core microbiota as the families that were present in 100% of the naive snails. Applying this definition, we identified 62 out of 97 families found in all naive individual snails (B0 and B25) and thus constituting the *B. glabrata* core-microbiota (Tables S5 and S9).

### 3.3. Microbiota Dynamics Following B. glabrata Infections by S. mansoni

After studying naive *Biomphalaria* bacterial microbiota, we investigated whether *Schistosoma mansoni* infections may affect the snail microbiota composition, structure and dynamics.

To investigate the influence of parasite infections on the bacterial microbiota, we analysed microbiota dynamics following sympatric or allopatric primary infections and homologous or heterologous secondary challenges (Figure 1).

**Figure 3:**
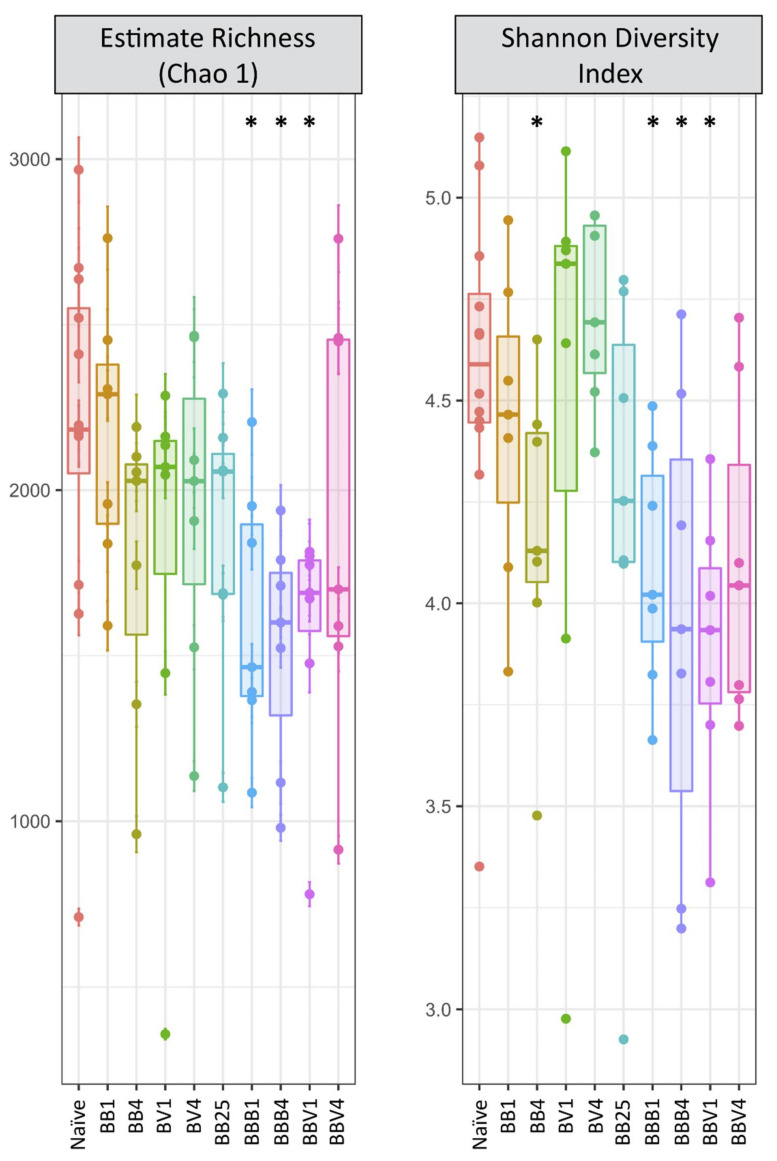
Microbiota alpha Diversity: Boxplots of Chao1 and Shannon indices for all samples. For the Naive condition, B0 and B25 snails were pooled; BB: primary infection of BgBRE by SmBRE; BV: primary infection of BgBRE by SmVEN; BBB: primary infection of BgBRE by SmBRE and secondary challenge by SmBRE; BBV: primary infection of BgBRE by SmBRE and secondary challenge by SmVEN. The time point is mentioned with 1, 4 or 25 corresponding to the day after primary infection or secondary challenge. The differences between naive and infected conditions were tested with a Mann–Whitney U test and significant differences mentioned with *.

We did not observe any significant changes in alpha diversity during primary infection compared to naive snails, except a significant decrease in Shannon’s H diversity index at day 4 after sympatric infection (Figure 3, Table S3, Mann–Whitney U test: *p* = 0.0251, Table S4). Conversely, all indices changed significantly following homologous or heterologous secondary challenges (Figure 3, Tables S3 and S4). Indeed, the observed species richness (Mann–Whitney U test, *p* = 0.0015), the Chao 1 richness index (Mann–Whitney U test, *p* = 0.0069), Shannon diversity index (*p* = 0.0008) and Pielou evenness index (Mann–Whitney U test, *p* = 0.0108) were significantly reduced following homologous or heterologous secondary challenges compared to naive and primary infected snails (Figure 3, Tables S3 and S4). However, the observed drop in alpha diversity disappeared by day 4 after the heterologous secondary challenge (Figure 3, Table S4). In addition, alpha diversity was mainly affected by the secondary challenge, regardless of it is homologous or heterologous. The primary infection did not significantly affect alpha diversity except for the sympatric combination on day 4 after infection (Mann–Whitney U test, *p* = 0.0251, Table S4).

**Figure 4:**
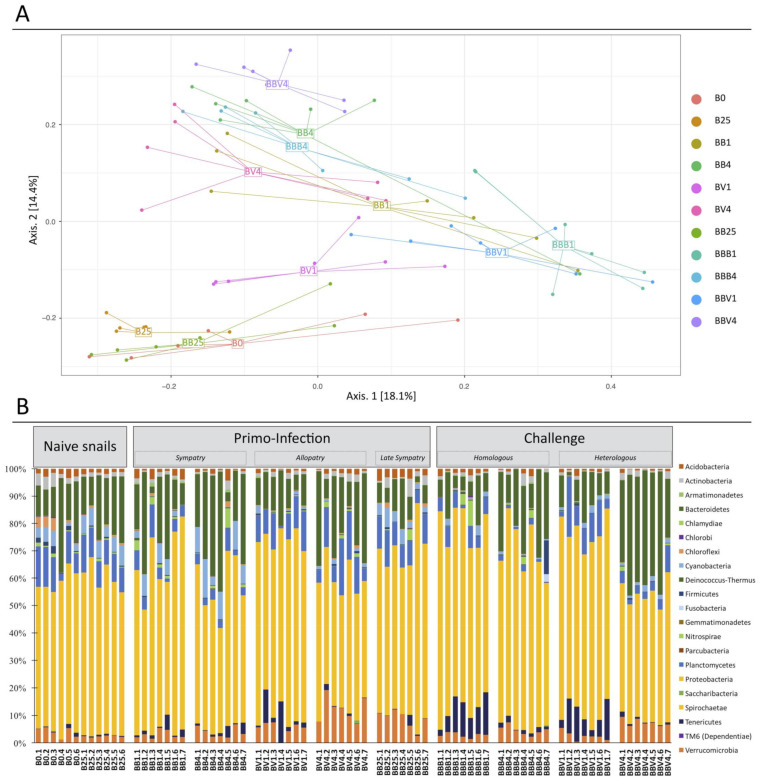
Beta diversity and bacterial communities following *Biomphalaria* infection: Dynamics of the bacterial microbiota of *Biomphalaria glabrata* following *Schistosoma* primary infection and secondary challenge. (**A**). Functional diversity comparisons of *Biomphalaria* microbiota along the infection. Principal coordinate analysis of pairwise Bray–Curtis distances across all infection type and time samples. Axes represent the two synthetic variables explaining the greatest proportion of variation in the data set. The sample name indicated in the figure corresponds to the centroid of all the biological replicates points of the respective experimental sample. (**B**). Phylum level composition of the 20 most abundant OTUs among all points of the kinetic. In this representation, the replicate naive snails were pooled for more readability.

Regarding the beta diversity, Principal Coordinate Analysis (PCoA) of Bray–Curtis dissimilarities revealed that BB25 samples grouped together with the naive snail samples (B0 and B25) and were separated from the infected snail samples along both axes (Figure 4A, Table S6). However, the BB25 samples were significantly different from the naive snail samples (Permanova Analysis and Post-Hoc Benjamini–Hochberg, *p* = 0.0023 and *p* = 0.0017, Table S6). The fact that BB25 grouped with naive snails rather than with infected-snail samples suggested that the snail microbiota is resilient to infection, with a tendency to recover between day 4 and day 25 after the primary infection (Figure 4A). Analysis of Bray–Curtis dissimilarity index revealed a significant difference between naive and primary infected samples (Permanova Analysis and Post-Hoc Benjamini–Hochberg, *p* = 0.001) and also between naive and secondary challenged samples (Permanova Analysis and Post-Hoc Benjamini–Hochberg, *p* = 0.001) (Table S6). Concerning the infected-snail samples, all experimental samples were significantly different from each other with the exception of BB1 versus BB4 and BBB1 versus BBV1 (Table S6).

In addition, the second PCoA axis separated the samples according to the course of infection (i.e., the day 1 from the day 4 samples) (Figure 4A), whereas the day 1 after-secondary challenge (BBB1, BBV1), the day 1 after primary infection (BB1, BV1) and all of the day 4 infection samples (BB4, BV4, BBB4, BBV4) were separated along the first axis (Figure 4A). Interestingly, bacterial communities on day 1 after the secondary challenge (BBB1, BBV1) were more different from the naive communities than the day 1 after primary infection (BB1, BV1). Moreover, the second axis separated the sympatric primary infection (BB1) from the allopatric primary infection (BV1) (Figure 4A). Finally, all day 4 samples grouped together reflecting the similarity between these samples regardless of experimental infection conditions (Figure 4A). Even if the day of infection appeared as the main explaining factor, these results indicated that the snail microbiota profile following infections depends on the nature of the immunobiological interactions (sympatric vs. allopatric or primary infection vs. secondary challenge).

**Figure 5:**
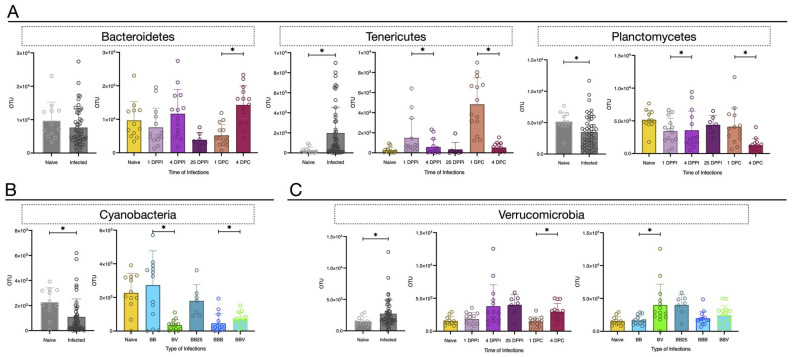
Dynamic of Specific Phylum during *Biomphalaria* infection: Dynamic of the number of OTU for specific Phylum according to 2 parameters. The time of infections: Day 1 (1 DPPI (=Day Post Primary Infection)) vs. Day 4 (4 DPPI) Primary infection and Day 1 (1 DPC (= Day Post-Secondary challenge)) and Day 4 (4 DPC) Secondary challenge. The type of infections: Sympatric (BB) vs. Allopatric (BV) Primary infections and Homologous (BBB) vs. Heterologous (BBV) Secondary challenge. (**A**). Phylums influenced by the time of infections. (**B**). Phylum influenced by the type of infections. (**C**). Phylum influenced by both time and type of infections. The Mann–Whitney U test is used to test the significant difference between the time or the type in Primary infections and Secondary challenge and mentioned with *.

We further investigated the microbial community dynamics following infection, and we observed some consistent changes in response to the various experimental infections tested (Figure 4B and Figure 5, Tables S7 and S10). Some phyla seemed to be influenced by the time of primary infections and/or secondary challenges (Figure 5A). This was the case for example for the Bacteroidetes (Figure 5A, Table S7) which relative abundance decreased 25 days after primary infections (Mann–Whitney Analysis, *p* = 0.0049) and increases significantly between 1 and 4 days after the secondary challenge (Mann–Whitney Analysis, *p* = 7.822.10^−5^). The relative abundance of Tenericutes (Figure 5A, Table S7) increased after primary infection (Mann–Whitney Analysis, naive vs. BB4 *p* = 0.001/naive vs. BV *p* = 0.001) and after the secondary challenge (Mann–Whitney Analysis, naive vs. BBB4 *p* = 0.0002/naive vs. BBV4 *p* = 0.001), and decreased significantly between 1 and 4 days after primary infection (Mann–Whitney Analysis, *p* = 0.021) or secondary challenge (Mann–Whitney Analysis, *p* = 1.994.10^−7^). We found that the Planctomycetes (Figure 5A, Table S7) significantly increased during the primary infections (Mann–Whitney Analysis, *p* = 0.021) but interestingly significantly decreased between day 1 and 4 after secondary challenges (Mann–Whitney Analysis, *p* = 0.0006).

Some other phyla seem to be more influenced by the type of parasites used for primary infection and/or secondary challenge (Figure 5B, Table S7). It is the case for the Cyanobacteria, for which we observed a significant decrease in the allopatric infection compared to the sympatric one (Mann–Whitney Analysis, *p* = 0.004). Interestingly, during the second challenge, we observed also a significant difference between the homologous and heterologous secondary challenge but in the opposite way. Indeed, the Cyanobacteria relative abundance was higher in the heterologous secondary challenge than the homologous one (Mann–Whitney Analysis, *p* = 0.04).

Finally, other phyla can be influenced by both time and type of infections (Figure 5C, Table S7). It is the case for the Verrucomicrobia, for which we observed a significant increase in the allopatric infection compared to the sympatric one (Mann–Whitney Analysis, *p* = 0.0014). In opposite, we observed a significant increase of these bacteria 4 days after the secondary challenge compared to 1 day (Mann–Whitney Analysis, *p* = 0.0016).

To conclude, depending on the phylum, we observed the largest modifications in bacterial microbiota composition influenced by the time of infection (day 1 and 4) after primary infections (sympatric/allopatric) and/or secondary challenges (homologous/heterologous).

Finally, we examined the core microbiota dynamics during infection. Similarly to the full microbiota, the core-microbiota, consisting of 62 families, was affected by the type of infection (naive vs. primary infection (Permanova Analysis and Post-Hoc Benjamini–Hochberg, *p* = 0.003) and naive vs. secondary challenge (Permanova Analysis and Post-Hoc Benjamini–Hochberg, *p* = 0.003)), by the time of infection (early (1 day) vs. late (4 days)) (Permanova Analysis and Post-Hoc Benjamini–Hochberg, *p* = 0.006) and also by the strain of parasite used for primary infection (SmBRE vs. SmVEN) (Permanova Analysis and Post-Hoc Benjamini–Hochberg, *p* = 0.016) (Table S8). Principal Coordinate Analysis (PCoA) based on Bray–Curtis dissimilarities between the core microbiota (Figure S2) yielded very similar results to those based on the entire dataset (Figure 4A).

In addition, we observed that 69.4% (43 families) of the core microbiota families were significantly affected by infection. Among those, 6.5% (4 families) were affected regardless of the infection type (Table S9). Interestingly, these families belong to the most abundant ones (Table S5). Nineteen families (30.6%) were never affected by infection (Table S9). Those families belong to the seldom-represented ones, except *Xanthomonadaceae,* which was the 7th most represented family (see Table S5). Further, 32.6% (14 families) of the core microbiota changed exclusively following primary infection and 4.7% (2 families) to secondary challenge (Table S9). Similarly, 20.9% (9 families) of the core microbiota were affected early after infection (1 day) and 7% later (3 families, day 4). Finally, 23.3% (10 families) were affected by the SmBRE infection and 9.3% (4 families) by the SmVEN infection (Table S9).

### 3.4. Link between the Microbiota Dysbiosis and B. glabrata Antimicrobial Immune Response

**Figure 6:**
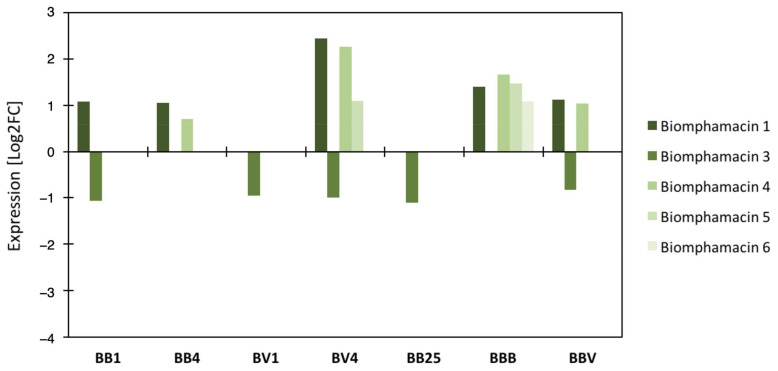
Differential gene expression of Biomphamacin antimicrobial peptides: Log2FC (fold change) of antimicrobial immune transcripts between naive and infected snails inferred from previous RNAseq analysis on the same experiment. A positive Log2 fold-change indicates over-expression in infected snails compared to the naive snails. Antimicrobial peptide families included 6 Biomphamacins (macin-like AMPs) consisting of 6 genes (shade of green).

The expression level of transcripts encoding antimicrobial peptides and antimicrobial proteins (AMP) was investigated following sympatric or allopatric primary infections and homologous or heterologous secondary challenges using RNAseq data (Figure 6 and Figure S3). Based on the *Biomphalaria glabrata* genome annotation [46] we identified 2 achacin genes, 5 lipopolysaccharide-binding protein/bactericidal permeability-increasing protein (LBP/PBI) genes, and 5 biomphamacin genes. LBP/BPI 3.1 and 3.2 were over-expressed at day 25 after primary infection and following the secondary challenges compared to naïve snails, while all other genes of this family were under-expressed in all infection conditions (Figure S3). The achacins were under-expressed following primary infection in sympatric combination, at day 25 after primary infection and following both secondary challenges (Figure S3). However, no differential expression was observed following allopatric primary infection compared to naive snails (Figure S3). Finally, the AMP biomphamacins 1, 4, 5 and 6 were over-expressed throughout the infection experiment, excepted for BV1 and BB25 (Figure 6). The biomphamacin 3 was mainly under-expressed except BV4 and BBB for which no differential expression was observed compared to naive snails (Figure 6).

Based on our observations of bacterial community shifts, changes in expression of antimicrobial molecules were expected to occur following primary infections and secondary challenges and not for BB25, where the resilience of the bacterial community has been observed (Figure 4 and Figure S2). In this context, the LBP/BPI and achacin seem not to be associated with microbiota changes observed herein as both remained highly under-expressed even at BB25 where microbial communities have already recovered (Figure S3). All biomphamacins, excepted the biomphamacin 3, were over-expressed after infection, from day 1 in BB infection and day 4 in BV infection and for homologous or heterologous secondary challenges (BBB and BBV). Finally, only a subset of biomphamacins (1, 4, 5, 6) changed in the course of infection but was not differentially expressed at BB25 compared with the naive snails (Figure 5), suggesting their possible link with microbiota dysbiosis.

## 4. Discussion

Fine-tuned interactions between microbiota, host immunity and pathogens have been observed in many vertebrate and invertebrate models [6,11,12,13,20]. Indeed, numerous studies revealed a tight control of the immune system on microbial community structure or composition, but further works are needed to clarify the link between microbiota and host immunity.

Herein, we investigated the interactions between the host immune system, parasite and the bacterial microbiota in an invertebrate model—the gastropod snail *Biomphalaria glabrata* and its trematode parasite *Schistosoma mansoni*. Depending on the past evolutionary history between snails and schistosomes, different immune responses against *S. mansoni* have been observed. We showed recently that in a sympatric interaction, the parasite that coevolved with its host induced a strong immunosuppression, whereas an allopatric interaction resulted in a strong host cellular immune response [31]. Moreover, a cellular immune response was observed following primary infection, but a humoral immune response was observed following homologous or heterologous secondary challenges [32]. So, using appropriate host-parasite combinations, we have the opportunity to modulate the host immune response to observe a functional and/or a composition alteration of the bacterial community of the snail reflecting a strong microbiota disequilibrium or dysbiosis [21,22,48]. First, we demonstrated that rearing conditions and water microbial composition do not affect snail bacterial microbiota composition and that this microbiota was highly specific with a limited inter-individual variation within mollusc strains or species. Then, studying the global bacterial microbiota community of *Biomphalaria* snails, we showed that the bacterial alpha diversity did not change following primary infection whatever the parasite strain or the timepoint of infection (Figure 3, Table S3). A decrease in alpha diversity was observed exclusively following secondary challenge infections, as reflected by multiple indices (Figure 3, Table S3). Conversely, primary infection and secondary challenge strongly affected the bacterial OTU composition (Figure 4). Moreover, differences in immunobiological interactions (immunosuppression, immune cellular response or immune humoral response) resulted in different microbiota dynamics reflected by specific changes in the snail microbial communities (Figure 4). Interestingly, homologous and heterologous secondary challenges activated a similar humoral immune response [31,32] and resulted in a similar change in microbiota alpha diversity and composition (Figure 3 and Figure 4, Table S3). Then, four days after infection, regardless of its type, the microbiota was still very different from the microbiota of naive snails, but the differences between the primary infection and secondary challenge disappeared, as apparent from the grouping of BB4, BV4, BBB4 and BBV4 in the PCoA (see Figure 4). Furthermore, most of the OTUs affected by the primary infection, returned to their initial state by day 25, indicating that the snail bacterial microbiota was resilient a few weeks after the infection (Figure 4). However, some differences persisted: the Verrucomicrobia phylum remained highly represented in the infected snails at day 25 after primary infection (Figure 4B and Figure 5C). Interestingly, some Verrucomicrobia species have been recently proposed as a hallmark of a healthy gut due to their anti-inflammatory and immune-stimulant properties and their ability to improve gut barrier function in human model [49]. The high abundance of Verrucomicrobia in the recovered snails could thus potentially reflect their role in community restoration (Figure 4 and Figure 5C). Moreover, the expansion of a Verrucomicrobia species *Akkermansia muciniphila* has been described in the gut of *S. mansoni*-infected mice, suggesting a potential functional role of Verrucomicrobia in *Schistosoma* infection processes in both definitive and intermediate hosts [50]. We also paid particular attention to the core microbiota. Given the various ways to define core microbiota, we considered exclusively the persistent occurrence in the bacterial community of naive *Biomphalaria glabrata* snails [51]. Similarly to the entire microbiota, the core microbiota was affected by the type of infection (naive vs. secondary challenge), by the time of infection (day 1 vs. day 4) as well as by the parasite strain (SmBRE vs. SmVEN) (Table S6). Core microbiota seemed to be affected by immunosuppression or the activation of the immune response in a similar way to the whole microbiota (Figure S2 and Figure 4A). Understanding the shifts in core microbiota following infection is important as the long-term stability and persistent occurrence of beneficial microbes and their associated functions may contribute to host health and homeostasis to maintain functionality and fitness toward changing ecological environments or environmental stress [52,53,54].

Previous microbiota analysis of *Biomphalaria glabrata* was performed a few years ago, but most of them focused on a cultivable part of the bacteria [33,34]. A more recent study using 16S rDNA amplicon sequencing compared the composition of bacterial microbiota between resistant and susceptible *Biomphalaria* phenotypes [38]. In our analysis, we also found most of the genera already identified excepted *Cupriavidus* and *Micavibrio*, but they corresponded to only 1.5% of all the bacterial community found in whole naive snail (Figure S4A). Even if all these genera were not the main part of the *Biomphalaria* microbiota, we observed a significant change during the *Schistosoma mansoni* infections (Figure S4B–D).

Given that infections affected the total and core microbiota composition, we explored the antimicrobial immune response following infection to find potential molecular mechanisms involved in the observed dysbiosis (Figure 6 and Figure S3). Among antimicrobial peptides (AMP) and antimicrobial proteins, the expression levels of AMP belonging to biomphamacin family were disturbed by the infections according to the time and the types of parasites (Figure 6). Since the AMPs are often considered as the main immune pathway responsible for bacterial microbiota regulation [55], an overall modification of the immune system may also be considered as a cause of dysbiosis. In our model, even though antimicrobial families can be involved in the complex process of regulation of microbiota communities, solely the biomphamacins AMP family members specifically displayed an expression pattern that can be linked with the observed dysbiosis of *Biomphalaria* bacterial communities (Figure 6 and Figure S3). Other pathways may play a potential role in the microbiota regulation like ROS/NOS pathways [56,57,58,59] and this will deserve further investigations in the present biological experimental model.

It has been shown that the immune system is a key determinant of host-associated bacterial communities in many biological systems. Two mechanisms have been proposed to explain host-microbiota interactions through crosstalk with the host innate immune system. The first one proposes that the host immune system exerts constant pressure on the microbiota to maintain homeostasis [60], the host immune system can thus control the composition of the resident microbiota [61]. According to this hypothesis, any changes in host immune response to infection would potentially affect the resident microbiota diversity and composition. The second mechanism proposes that the immune system would be tolerant to weak and continuous antigenic immune stimulations experienced during a lifespan [62], and thus that the host immune system would not exert any pressure or control on the resident microbiota. Based on this second hypothesis, if the immune system is not involved in the control of microbiota, thus any changes in host immune response to infection would potentially not affect the resident microbiota diversity and composition.

However, numerous studies demonstrated a direct control of microbial communities by the host immune system. As an example, species-specific antimicrobial peptides can shape species-specific bacterial associations in *Hydra* [63]. Other immune pathways have been also demonstrated to regulate or control the microbiota communities, like the intestinal homeo-box gene Caudal in *Drosophila* [15], or neuropeptides with an antibacterial activity which are secreted to shape the microbiota on the body surface of *Hydra* [64], even host lectins were demonstrated to stabilize microbiota communities [65].

Thus, if biotic stress (i.e., an infection) modifies the expression of antimicrobial peptides or other immune-related pathways, an effect on the microbial communities can be expected. Our results indicated that bacterial communities could indeed be shaped by the immune system of *B. glabrata*. Based on the hypothesis proposed by Hooper and collaborators [60,65,66], it seems that the immune system of *B. glabrata* snails maintains or controls the microbial communities permanently. Therefore, following an infection, the immune system is diverted from its function of managing the microbiota thus consequently releasing its control on the bacterial communities, resulting in changes in their composition and diversity. When the immune response returns to a basal level, the microbiota then returns to its “healthy” state [60,66], as observed in the present study (Figure 4). In other words, the immune system is likely no longer able to maintain the microbiota homeostasis after infection (resulting in dysbiosis), which, may in turn affect host homeostasis or fitness [67,68].

In an interaction between *Biomphalaria glabrata* and *Schistosoma mansoni* that results from a long co-evolutionary history, we can’t omit the parasite role. Indeed, the parasite has co-evolved with the host and so with its microbiota and could have been under selection pressures to manipulate the host microbiota or exploit the snail immunity to optimize host infection. This scenario has been already demonstrated for another invertebrate model, *Tenebrio molitor* which is infected by the tapeworm *Hymenolepis diminuta* [69]. In this interaction, the authors observed a significant decrease of parasite establishment after an antibiotic treatment, suggesting that the microbiota contributes to the parasite worm infection or indirectly by modification of the host immune system due to antimicrobial treatment and modification of the microbiota.

To conclude, changes in microbiota composition may result from shifts in the abundance of specific bacterial groups participating in anti-pathogen response, or just be a collateral effect of the immune response activation against metazoan parasite infection, or may also be an active host’s manipulation by the parasite as a result of the long co-evolutionary history between both protagonists. These questions will deserve further investigations, by testing *S. mansoni* prevalence and intensity in experimental infections of *Biomphalaria glabrata* snails following antibiotic treatment or microbiota transplantation.

To fully understand Schistosomiasis transmission and to develop new strategies of control the expansion of this widespread human parasitic disease in the field, it will be crucial to determine if the snail-associated bacterial communities affect the parasite transmission. For example, expanding knowledge on *Biomphalaria* snail microbiota is an essential step for developing paratransgenetic solutions to the spread of Schistosomiasis, involving the use of transgenic bacteria expressing foreign gene products (i.e., schistosomicidal compounds) that can reduce host competence or block pathogen development or transmission when introduced into the microbiota of vector snail field populations [70,71,72]. Moreover, specific microbiota composition or alteration of the microbiota (dysbiosis) may change host susceptibility or competence towards pathogens. The microbiome could enhance or reduce snail competence by direct interaction with the parasite or by stimulation of the immune system [35]. Thus, it would be possible to identify markers of high transmission snails based on the diversity and composition of their microbiota. This characterization of snail’s microbiota in transmission foci would thus be used to predict epidemiological risks. As snail genotype constraint the microbiota composition and diversity [38], we can also propose a vector mediation strategy based on the introgression in transmission foci of selected snail genotypes associated with a specific microbiota refractory to schistosomiasis infection. Finally, instead of using molluscicides that are detrimental for the ecosystem, we can propose to use chemicals dedicated to modify the microbiome or microbiota in a way of reducing parasite infection and increasing snail resistance to the Schistosome parasite.

The present study therefore has to be considered as the first step, that will gives an overview of the microbiota diversity at intra-specific and inter-individual levels and of microbiota response to *S. mansoni* infection. Then, a wider program on the role of microbiota on snail immunity and compatibility with Schistosomes would be necessary to find crucial ways to influence the establishment success and eventually the transmission dynamics of Schistosomiasis diseases. All these approaches will now deserve further considerations to reduce Schistosomiasis outbreaks in the upcoming years.

## Data Availability

The data presented in this study are available on request from the corresponding author. The data are not publicly available due to ongoing analysis of the data and valorisation.

## Supplementary Materials

Materials could be found at https://www.mdpi.com/article/10.3390/microorganisms9051084/s1. Figure S1: Snail Microbiota and Water Microbial Communities; Figure S2: Beta Diversity of the Core Microbiota; Figure S3: Differential gene expression of LBP/BPI and Achacin antimicrobial protein families; Figure S4: Dynamic of Genera discovered in previous studies of the *Biomphalaria* microbiota; Table S1: List of OTU composition in metabarcoding 16S sequencing; Table S2: List of antimicrobial genes; Table S3: Alpha diversity of all experimental conditions; Table S4: Statistical analysis of alpha diversity; Table S5: List of OTU composition in Core Microbiota; Table S6: Statistical analysis of beta diversity; Table S7: Statistical analysis of phylum dynamic following infections; Table S8: Statistical analysis of core-microbiota dynamic; Table S9: Core microbiota dynamic; Table S10: Statistical analysis of dynamic of the microbiota genus.
