# Supplementary material for: Experimental Infection of the Biomphalaria glabrata Vector Snail by Schistosoma mansoni Parasites Drives Snail Microbiota Dysbiosis
Source: Microorganisms. 2021 May 18;9(5):1084. doi: 10.3390/microorganisms9051084 (PMC8158356; doi:10.3390/microorganisms9051084)

## Slide 1
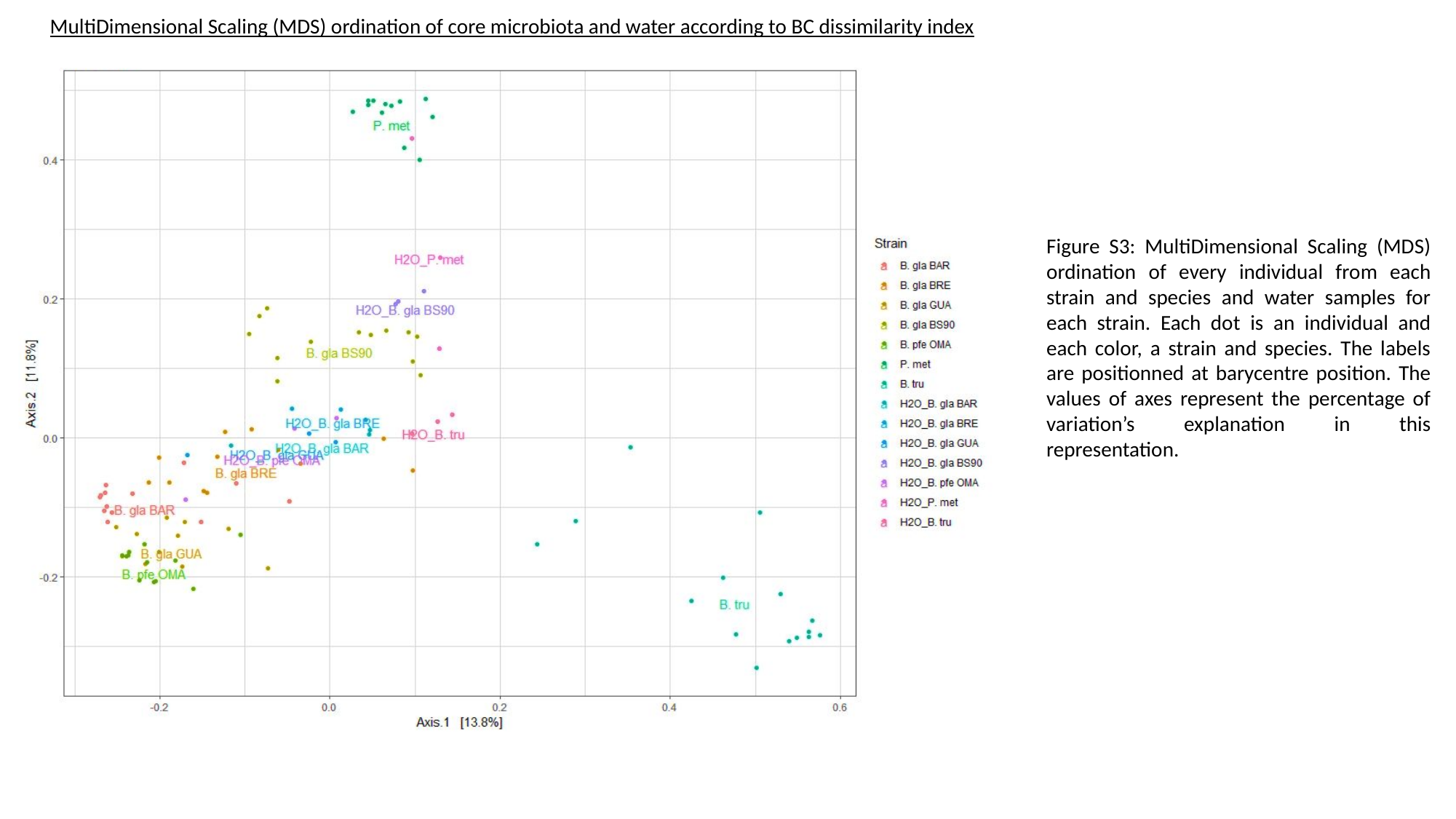

MultiDimensional Scaling (MDS) ordination of core microbiota and water according to BC dissimilarity index
Figure S3: MultiDimensional Scaling (MDS) ordination of every individual from each strain and species and water samples for each strain. Each dot is an individual and each color, a strain and species. The labels are positionned at barycentre position. The values of axes represent the percentage of variation’s explanation in this representation.

## Slide 2
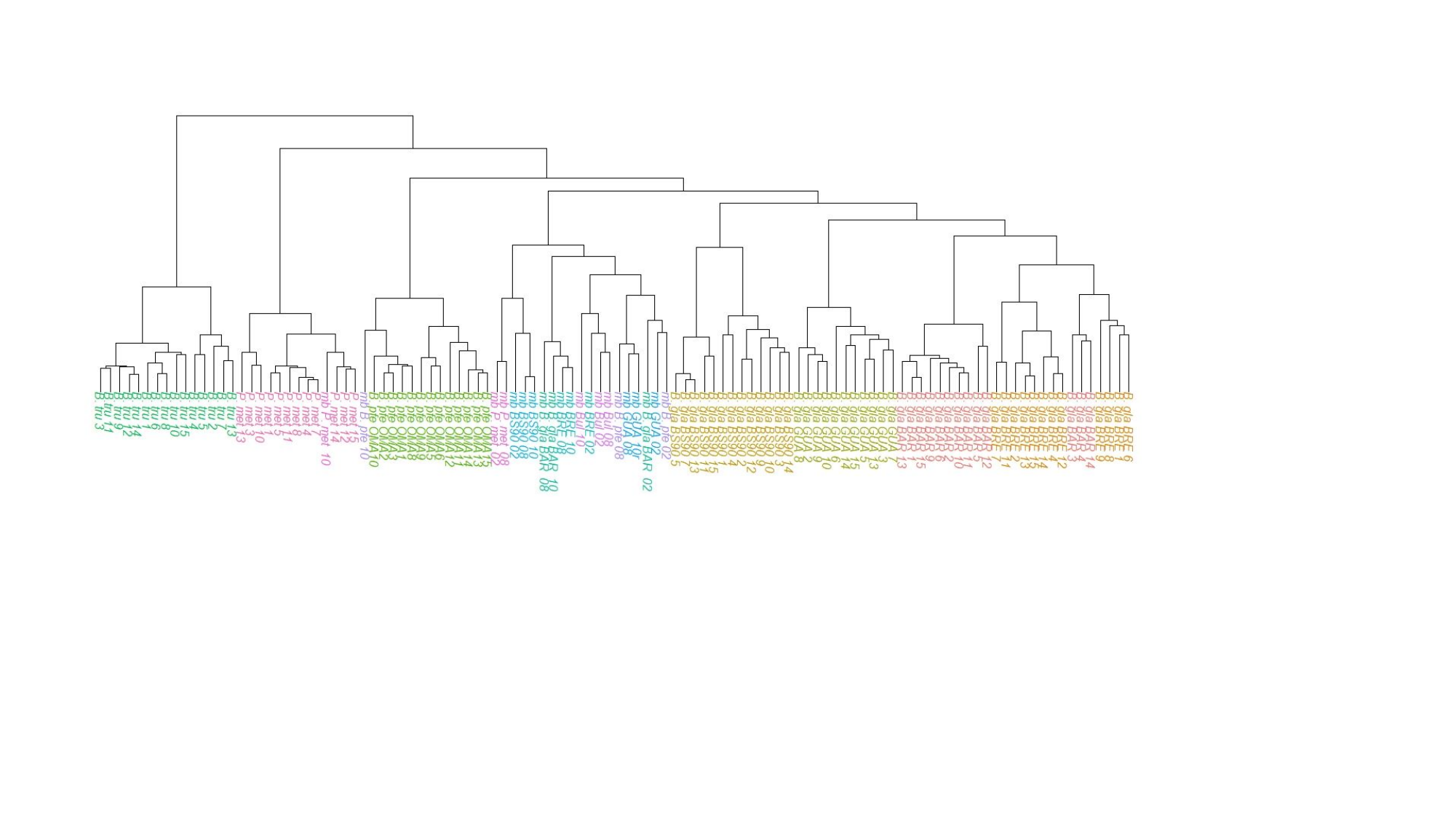

Supplement: Supplementary file 1 [file microorganisms-09-01084-s001.zip › Supp_files Microbiomph/Fig.S1_Snail_Microbiota_Water_Microbial_Communities.pptx]
