# Supplementary figures and images for: Experimental Infection of the Biomphalaria glabrata Vector Snail by Schistosoma mansoni Parasites Drives Snail Microbiota Dysbiosis
Source: Microorganisms. 2021 May 18;9(5):1084. doi: 10.3390/microorganisms9051084 (PMC8158356; doi:10.3390/microorganisms9051084)

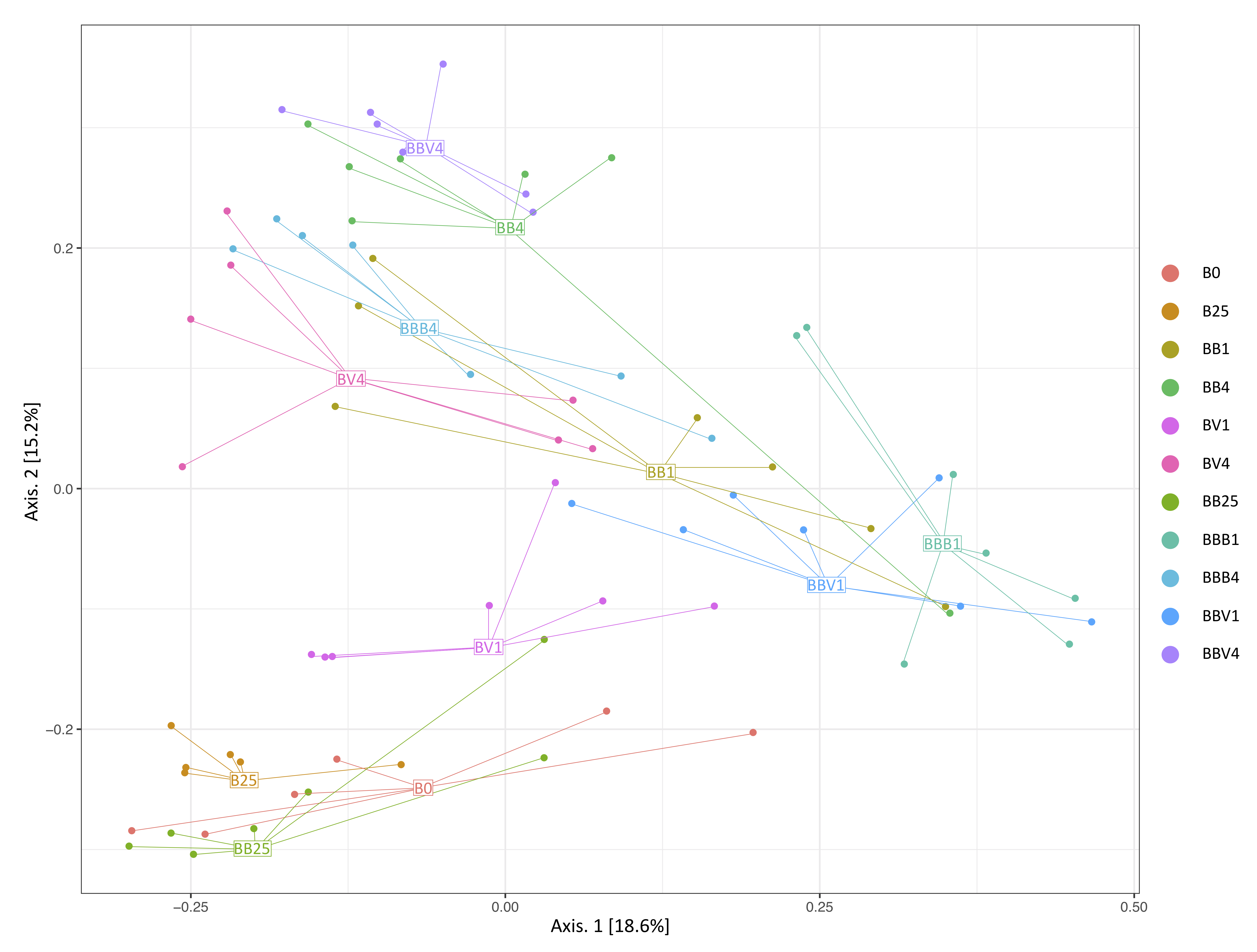

Supplement: Supplementary file 1 [file microorganisms-09-01084-s001.zip › Supp_files Microbiomph/Fig.S2_PCoA_Core_Microbiota.tiff]

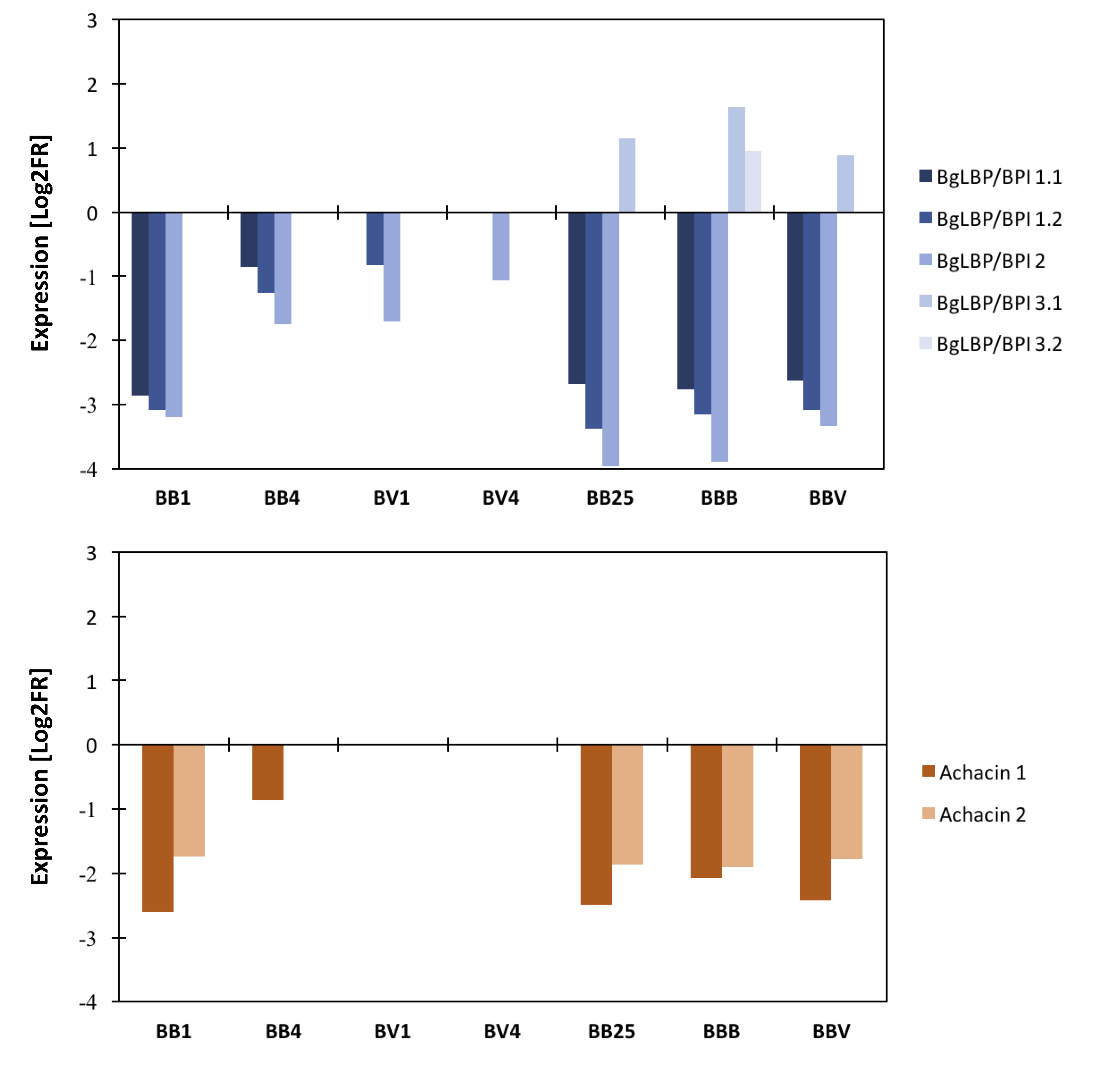

Supplement: Supplementary file 1 [file microorganisms-09-01084-s001.zip › Supp_files Microbiomph/Fig.S3_antimicrobial_immune_response.tif]

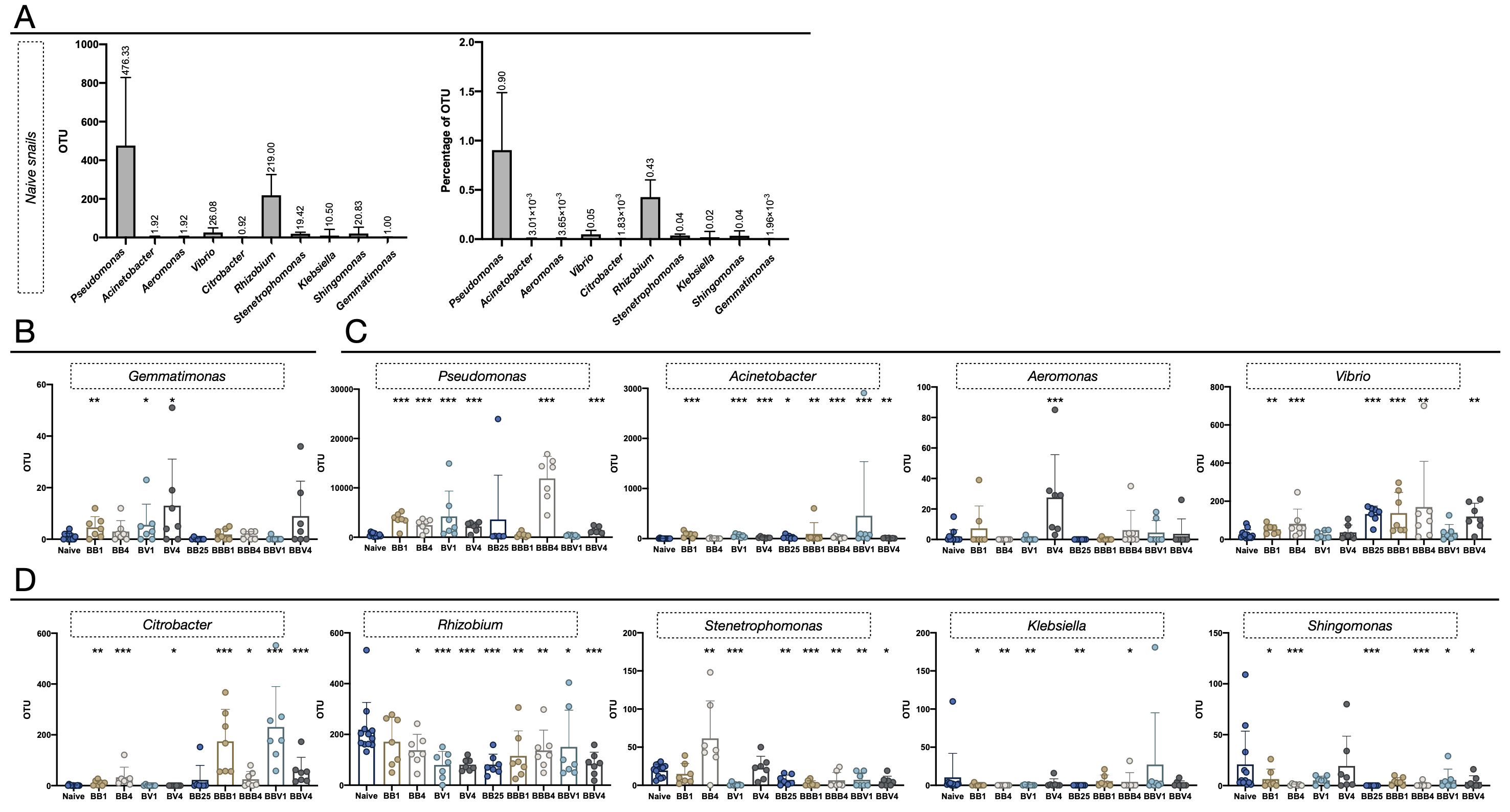

Supplement: Supplementary file 1 [file microorganisms-09-01084-s001.zip › Supp_files Microbiomph/Fig.S4_previous_results.tiff]
